# Editorial: Gut-brain axis correlates, mediators, and moderators of stress resilience or vulnerability

**DOI:** 10.3389/fnbeh.2025.1629472

**Published:** 2025-06-17

**Authors:** Annelise A. Madison, Silvia Turroni, Audrey F. Duff, Jeff D. Galley, Amy R. Mackos, Avinash Veerappa

**Affiliations:** ^1^Department of Psychology, University of Michigan, Ann Arbor, MI, United States; ^2^Department of Pharmacy and Biotechnology, University of Bologna, Bologna, Italy; ^3^Center for Microbe and Immunity Research, Abigail Wexner Research Institute at Nationwide Children's Hospital, Columbus, OH, United States; ^4^The Ohio State University Medical Center, Columbus, OH, United States; ^5^College of Nursing, The Ohio State University, Columbus, OH, United States; ^6^Department of Genetics, Cell Biology and Anatomy, University of Nebraska Medical Center, Omaha, NE, United States

**Keywords:** gut-brain axis, microbiota, neurodegenerative, Mendelian Randomization study, resilience, stress, endotoxemia

Recognizing the wide variability in stress responses and resilience, we invited studies extending emerging evidence that the gut microbiota mediates or moderates these responses. Submissions ranged from infancy to old age, short- to longer-term, and from small cohorts to population studies. We knew this call would be challenging: the gut-brain axis literature suffers from inconsistent methods, limiting translation and reproducibility. Many paradigm-shifting findings among animals have not translated to humans. Still, diverse exploratory and confirmatory studies are vital to pinpoint when and how gut-brain communication occurs. Such research is methodologically complex, necessitates expertise across disciplines. Here we present findings from five research teams who pursued this cross-cutting research in humans.

Night shift work is a naturalistic stressor that can profoundly disrupt the circadian rhythm. Yao et al. used a longitudinal design among 10 psychologically healthy clinicians across ≥4 consecutive night shifts. They collected actigraphy data as well as three functional magnetic resonance imaging (fMRI) assessments (pre-shift, post-shift, and recovery), which were preceded by fecal samples and followed by a cognitive battery. From stool, they assessed the microbiome and short-chain fatty acids (SCFAs). On average, participants lost over an hour of sleep during their shifts; however, cognitive test performance, the microbiome, and SCFAs were unchanged over time. fMRI data revealed significant functional changes in the superior frontal gyrus and reduced connectivity between key brain regions (e.g., posterior cingulate cortex and thalamus). In contrast, control participants exhibited functional stability. Although microbiota diversity did not correlate with brain functional connectivity, the association between the relative abundance of certain phyla and key brain region connectivity reversed during shift work, indicating altered gut-brain communication.

Another interesting approach is to use plasma or serum indicators of gut barrier permeability, which can affect immune function. McDonnell et al. used longitudinal data from a racially diverse sample of 162 healthy adults, showing that baseline levels of endotoxemia predicted working memory improvement across the next 9 and 18 months, perhaps indicative of practice effects; however, those with higher endotoxemia levels at baseline did not show these improvements. Cross-sectionally, men—but not women—with higher levels of endotoxemia also performed better on the working memory task. The authors speculated that this unexpected finding may have resulted from increased alertness via endotoxin stimulation of alpha and beta waves. They also asserted that endotoxemia likely does not benefit males in the long-term, as their prospective models demonstrate. Further work should explore the relationship between endotoxemia, brain function, and cognition across varying timescales.

Three of our studies used Mendelian Randomization (MR) to understand the directionality of the relationship between the gut microbiota and neurological disease. This approach allows for causal inference given genetic variations are the instrumental variables underlying the exposure of interest (i.e., the microbiome). However, it is vital to include additional layers of evidence to support these findings, considering the multifactorial determinants of microbiome composition that extend beyond genetics. The MR approach using microbiome data is still in its early stages. However, the three studies discussed below contribute to the growing evidence that gut microbiota may causally influence neurological diseases development, and that brain structure and gastrointestinal function may mutually affect each other. Although these MR studies focus on disease risk rather than stress responses, they offer insight into long-term consequences of gut-brain interactions.

Two MR studies, Wang et al. and Li et al. utilized the prior genome-wide association analysis (GWAS) from the MiBioGen collaboration, which includes >18,000 people primarily of European descent (Kurilshikov et al., 2021). Wang et al. also utilized the GWAS of six neurodevelopmental outcomes (cerebral palsy, intellectual disability, anxiety neurosis, autism, behavioral and emotional disorders, and attention deficit hyperactivity disorder) from FinnGen to examine whether the microbiome was causally associated with neurodevelopmental outcomes in preterm infants. Li et al. used MiBioGen data to explore relationships between the microbiome and seven common neurological diseases in adults: epilepsy, schizophrenia, bipolar disorder, Alzheimer's disease, Parkinson's disease, brain cancer, and stroke. Both MRs were taxonomically comprehensive, spanning phylum to genus, and both found risk-related and protective microbiota taxa. Although these authors proposed potential causal interpretations, lack of multiple testing correction significantly limits the strength of such inferences. Nonetheless, these studies highlight several associations that merit further validation in future hypothesis-driven research.

In the last MR study, which utilized multiple test corrections, Xu et al. investigated bidirectional causal pathways between cerebral cortex structure and functional gastrointestinal disorders (FGIDs), such as irritable bowel syndrome (IBS), using data from the FinnGen and ENIGMA databases. Importantly, their analyses also accounted for anxiety and depression, which commonly co-occur with FGIDs. Functional dyspepsia was causally associated with reduced cortical thickness of the rostral anterior cingulate cortex (rACC) and a greater surface area of the caudal anterior cingulate cortex (cACC) was causally associated with IBS. As the authors point out, the cACC is involved in pain processing and responding. Greater pain sensitivity may increase stress and affect symptom appraisal, thereby paving the way for IBS diagnosis. Notably, anxiety and depression did not mediate these relationships, suggesting a direct, causal relationship between rACC/cACC structure and FGIDs.

Collectively, these findings present foundational evidence that night shift work may acutely perturb relationships between gut microbiota taxa abundance and brain functional connectivity, and that over a much longer time period, gut barrier permeability may track with diminished practice effects on subsequent working memory tests among healthy people. In terms of risk for stress-related diseases or disorders, ACC structure and FGIDs may have a bidirectional, causal relationship (independent of anxiety and depression), and gut microbiota composition may increase risk or protect against neurological diseases in preterm infants and adulthood. Because these studies are among the first of their kind, more replication is needed before clinical application of these findings. In the future, bioinformatics (e.g., non-linear methods), artificial intelligence, and integrative systems biology may help to cohere and advance this promising literature.
